# Activation of 5‐HT_2A_ receptors by TCB‐2 induces recurrent oscillatory burst discharge in layer 5 pyramidal neurons of the mPFC in vitro

**DOI:** 10.14814/phy2.12003

**Published:** 2014-05-20

**Authors:** Michael S. Spindle, Mark P. Thomas

**Affiliations:** 1 School of Biological Sciences, University of Northern Colorado, Greeley, Colorado

**Keywords:** 5‐HT2A, layer 5 pyramidal neuron, mPFC, serotonin, TCB‐2

## Abstract

The medial prefrontal cortex (mPFC) is a region of neocortex that plays an integral role in several cognitive processes which are abnormal in schizophrenic patients. As with other cortical regions, large‐bodied layer 5 pyramidal neurons serve as the principle subcortical output of microcircuits of the mPFC. The coexpression of both inhibitory serotonin 5‐HT_1A_ receptors on the axon initial segments, and excitatory 5‐HT_2A_ receptors throughout the somatodendritic compartments, by layer 5 pyramidal neurons allows serotonin to provide potent top–down regulation of input–output relationships within cortical microcircuits. Application of 5‐HT_2A_ agonists has previously been shown to enhance synaptic input to layer 5 pyramidal neurons, as well as increase the gain in neuronal firing rate in response to increasing depolarizing current steps. Using whole‐cell patch‐clamp recordings obtained from layer 5 pyramidal neurons of the mPFC of C57/bl6 mice, the aim of our present study was to investigate the modulation of long‐term spike trains by the selective 5‐HT_2A_ agonist TCB‐2. We found that in the presence of synaptic blockers, TCB‐2 induced recurrent oscillatory bursting (ROB) after 15–20 sec of tonic spiking in 7 of the 14 cells. In those seven cells, ROB discharge was accurately predicted by the presence of a voltage sag in response to a hyperpolarizing current injection. This effect was reversed by 5–10 min of drug washout and ROB discharge was inhibited by both synaptic activity and coapplication of the 5‐HT_2A/2C_ antagonist ketanserin. While the full implications of this work are not yet understood, it may provide important insight into serotonergic modulation of cortical networks.

## Abstract (precis)

Using whole‐cell patch‐clamp recordings obtained from layer 5 pyramidal neurons of the mouse mPFC, we investigated the modulation of long‐term spike trains by the selective 5‐HT_2A_ agonist TCB‐2. In the presence of synaptic blockers, TCB‐2 induced recurrent oscillatory bursting (ROB) after 15–20 sec of tonic spiking in 7 of the 14 cells; ROB discharge was accurately predicted by the presence of a voltage sag in response to a hyperpolarizing current injection. We have identified a novel modulation of pyramidal neuron excitability by a 5HT receptor known to contribute to the pathophysiology of schizophrenia.

## Introduction

The medial prefrontal cortex (mPFC) of rodents is an agranular cortex that consists of ventral (infralimbic and prelimbic), medial (orbital), and dorsal (precentral and anterior cingulate) regions (Heidbreder and Groenewegen 2003; Dalley et al. 2004). It has been proposed that the mPFC of rodents may be homologous to medial and lateral prefrontal cortex in humans and primates (Heidbreder and Groenewegen 2003; Seamans et al. 2008). The mPFC of rodents contains at least two distinct populations of layer 5 pyramidal neurons that display different morphological and physiological characteristics; these differences reflect their axonal projections with one group of neurons (thin‐tufted) projecting cortically and the other group (thick‐tufted) projecting subcortically (Christophe et al. 2005; Dembrow et al. 2010). Subcortically projecting neurons display a prominent sag in response to hyperpolarizing current injection, and a pronounced depolarizing afterpotential (DAP) immediately following action potentials. In addition, these neurons are capable of firing a relatively high‐frequency burst of action potentials in response to suprathreshold stimulation. Due to their electrophysiological differences (e.g., Yang et al. 1996), these neuron types are also referred to as intrinsically bursting (subcortically projecting) and regular spiking (commissurally projecting).

The mPFC is one of several regions of the brain which has been found to be anatomically and functionally abnormal in schizophrenic patients (Honea et al. 2005; Pomarol‐Clotet et al. 2010). This region is interesting because it has also been shown to underlie executive functions which are often disrupted in schizophrenia such as working memory (Delatour and Gisquet‐Verrier 1999), and attentional set shifting (Birrell and Brown 2000), as well as extinction of acquired conditioning (Morgan et al. 1993) and prepulse inhibition (for review see Braff 2010). Despite the knowledge that these regions are implicated in schizophrenic etiology, there is no clear understanding of the perturbations in activity at the local network or single‐cell level. As the primary output stage of cortical microcircuits, layer 5 pyramidal neurons likely play an integral role in mediating schizophrenic symptoms and the modulation of these neurons likely has major ramifications for global information processing.

Although schizophrenia research has typically focused on dopaminergic systems, it is important to examine the role of the serotonergic modulation of cortical networks in the mPFC. The efficacy of atypical antipsychotic drugs is thought to stem partly from their antagonism of serotonergic receptors (Meltzer 1994; Meltzer and Massey 2011); in addition the psychotomimetic effects of LSD and other psychedelic hallucinogens result from their activation of the excitatory 5‐HT_2A_ receptor (Glennon et al. 1984; Titeler et al. 1988). Although qualitatively different effects are observed in vivo, hallucinogenic drugs that activate 5‐HT_2A_ receptors produce some of the same abnormalities in network activity as NMDA receptor antagonists such as phencyclidine (see Aghajanian and Marek 2000) which is used to induce schizophrenic‐like symptoms in animal models (see Mouri et al. 2007).

Serotonergic neurons from the dorsal raphe nuclei innervate the mPFC and exert top–down control of information processing largely via nonsynaptic volume transmission (Seguela et al. 1989; Smiley and Goldman‐Rakic 1996). Serotonin (5‐HT) receptors are expressed throughout the cortex following a caudal–rostral gradient with the highest density in the medial prefrontal cortex (Weber and Andrade 2010) and can be found on both glutamatergic (Amargos‐Bosch et al. 2004) and GABAergic (Lee et al. 2010; Puig et al. 2010) neurons. Layer 5 pyramidal neurons have been found to exhibit excitatory, inhibitory, or biphasic responses following application of 5‐HT (Araneda and Andrade 1991; Avesar and Gulledge 2012) due to the coexpression of both the excitatory 5‐HT_2A_ and the inhibitory 5‐HT_1A_ receptors (Amargos‐Bosch et al. 2004). Activation of the G_q_‐coupled 5‐HT_2A_ receptor has been shown to increase the amplitude and frequency of excitatory postsynaptic potentials (EPSPs) (Aghajanian and Marek 1997). Serotonin‐2A receptor activation has also been found to alter neuronal firing behavior by increasing both steady‐state firing rate and firing rate gain in response to depolarizing current steps in layer 5 pyramidal neurons (Zhang and Arsenault 2005).

In addition to gain increases, activation of the 5‐HT_2A_ receptor has also been found to increase the probability of burst discharge in pyramidal neurons of the mPFC in rats in vivo (Celada et al. 2008), and in pyramidal neurons of the electrosensory lateral line lobe of the *Apteronotus leptorhynchus* in vitro (Deemyad et al. 2011). Rhythmically recurrent burst discharge has previously been reported as both an intrinsic property of deep layer pyramidal neurons of the mPFC (Yang et al. 1996), and an evoked response to concurrent dendritic and somatic stimulation (Schwindt and Crill 1999; Williams and Stuart 1999; Larkum et al. 2004).

In general, in vitro studies examine spiking behavior over relatively short periods of time (500–5000 msec). Because of this, relatively little is understood about the role of serotonin in the long‐term modulation of neuronal firing mode. The purpose of this study was to examine the effects of prolonged 5‐HT_2A_ receptor activation on the sustained firing properties of layer 5 pyramidal neurons throughout relatively long 60 sec DC current injections in vitro.

## Methods

### Tissue preparation

Young C57bl/6 mice (age 27‐ to 44‐day‐old, *n* = 29) of both sexes were used in this study. All procedures used in this study were approved by the University of Northern Colorado institutional animal care and use committee. Mice were anesthetized with CO_2_ and rapidly decapitated. Brains were rapidly removed and placed immediately in ice cold sucrose‐containing artificial cerebrospinal fluid (aCSF) which consisted of (in mmol/L): sucrose 206, NaHCO_3_ 25, dextrose 10, KCl 3.3, NaH_2_PO_4_ 1.23, MgCl_2_ 4, CaCl_2_ 1, osmolarity was adjusted to 295 ± 5 mOsm and pH was adjusted to 7.40 ± 0.03. Coronal sections of the prefrontal cortex were obtained with a vibrating tissue slicer (OTS‐5000, Electron Microscopy Sciences, Hatfield, PA). Sections were cut 300 μm thick and were taken from approximately 200–1400 μm caudal to the frontal pole. Slices were transferred to a holding chamber containing aCSF which was composed of (in mmol/L): NaCl 120, NaHCO_3_ 25, dextrose 10, KCl 3.3, NaH_2_PO_4_ 1.23, CaCl_2_ 2.0, MgCl_2_ 1.0, osmolarity was adjusted to 295 ± 5 mOsm and pH was bubbled to saturation with 95% O_2_–5% CO_2_ gas and if necessary adjusted to 7.40 ± 0.03. Throughout experiments aCSF was bubbled constantly with 95% O_2_–5% CO_2_ gas. Slices were incubated in the holding chamber at 34°C for 45 min and then held at room temperature for up to 4 h thereafter. For electrophysiology experiments, slices were transferred to a recording chamber which was constantly superfused with oxygenated aCSF at a rate of 1–2 mL/min. The recording chamber was held at a constant 33–35°C with a temperature controller (model TC‐344b, Warner Instrument Corp., Hamden, CT) throughout the duration of the experiments.

### Electrophysiology

Layer 5 pyramidal neurons of the infralimbic, prelimbic, and anterior cingulate cortices were visually identified via infrared DIC microscopy at 400× magnification with an Olympus BX51WI microscope (Tokyo, Japan). Whole‐cell recordings were obtained after the formation of a minimal 1 GΩ membrane seal. Only cells that exhibited thin, overshooting action potentials, and would consistently spike throughout a 60 sec current injection were used in this study. Access resistance (R_A_) was monitored and compensated throughout experiments, and cells were excluded from analysis if the uncompensated R_A_ exceeded 20 MΩ. During current injections, amplifier bridge balance was utilized and monitored throughout the experiment. Patch pipettes used in this study were produced from thin‐wall glass capillary tubes (1.5 μm OD, 1.12 μm ID, World Precision Instruments, Sarasota, FL) with a Narishige PC‐10 pipette puller (Narishige, Tokyo, Japan). Pipettes were filled with an intracellular recording solution which contained (in mmol/L): potassium gluconate 135, KCl 10, EGTA 0.02, HEPES 10, MgATP 2, tris GTP 0.38. Recordings were obtained with an A‐M Systems model 2400 amplifier (A‐M Systems, Sequim, WA) and digitized with a Digidata 1322a DAC (Molecular Devices, Sunnyvale, CA). Data were acquired at a 10 kHz sampling rate using pClamp 8.1 software. Sag amplitude was assessed (mean amplitude = 2.8 mV) in response to a 150 pA hyperpolarizing current injection and was normalized as a percentage (minimal voltage/voltage at 350 × 100 msec). To assess relatively long‐term spiking characteristics in vitro, neurons were held at suprathreshold potentials by injection of DC current in bridge balance mode.

### Drugs

The selective 5‐HT_2A_ agonist TCB‐2 and 5‐HT_2A/2C_ antagonist ketanserin were purchased from Tocris Biosciences (Bristol, UK). To elucidate the effects of 5‐HT_2A_ activation on intrinsic neuronal properties irrespective of network activity, the GABA_A_ antagonists bicuculline methiodide (Enzo Life Sciences, Farmingdale, NY) and gabazine (Tocris, Bristol, UK) and the AMPA antagonist 6,7‐dinitroquinoxaline‐2,3‐dione (DNQX) (Alomone Labs, Jerusalem, Israel) were used either alone or together in most experiments. Bicuculline, gabazine, and TCB‐2 were each diluted into aliquots of 10 mmol/L stocks, DNQX was diluted to 20 mmol/L, and ketanserin was stored in 1.25 mmol/L aliquots. All drugs were stored at −80°C and then diluted to working concentrations of 10 μmol/L TCB‐2, 10 μmol/L bicuculline, 10 μmol/L gabazine, 20 μmol/L DNQX, and 750 nmol/L ketanserin in aCSF. Any drugs that were not used within 3 days of thawing were discarded. For electrophysiology experiments, brain slices were superfused with drug solutions for 5 min prior to any data acquisition.

### Data analysis

Axon binary files recorded in pClamp 8.1 were loaded into the MATLAB mathematical suite (Mathworks Inc., Natick, MA) and analyzed with custom scripts. Burst discharges in response to 60 sec depolarizing current injections were assessed by calculating the coefficient of variation (Cv, standard deviation‐to‐mean ratio) of interspike intervals (ISI). Since relatively long traces were recorded, the development of bursting behavior was assessed by calculating the Cv of the minimal voltage amplitude during the interspike intervals (ISI_min_) of a 6‐spike moving average throughout the trace. This method of calculating the Cv of ISI_min_ was used in this context due to its higher sensitivity to bursts over short periods of time. We defined burst initiation where ISI_min_(*n* + 1) − ISI_min_(*n*) >2 mV, and burst termination where ISI_min_(*n* + 1) − ISI_min_(*n*) <−2 mV. The threshold of ±2 mV was chosen based on its accuracy in predicting visually identified bursts with minimal false positives. Burst initiation and termination times were verified by visually inspecting data records. This method was used to assess the number of spikes per burst and the percentage of intraburst spikes relative to total evoked spikes.

## Results

Stable whole‐cell recordings were obtained from 40 layer 5 pyramidal neurons. The mean resting potential was −70.5 ± 5.4 mV (±denotes standard deviation), C_M_ 188.7 ± 56.4 pF, R_IN_ 105.0 ± 30.2 MΩ, and threshold to action potential −46.4 ± 4.0 mV. Application of 10 μmol/L TCB‐2 resulted in a mean depolarization of 4.3 mV (*n* = 7, control mean = −68.9 ± 5.9 mV, TCB‐2 mean = −64.6 ± 5.6 mV, *P* = 0.036, paired *t*‐tests), and a 15.1 MΩ mean increase in input resistance (*n* = 7, control mean = 98.5 ± 45.4 MΩ, TCB‐2 = 113.5 ± 46.7MΩ, *P* = 0.007, paired *t*‐tests).

### TCB‐2 induces burst discharge

**Figure 1.**
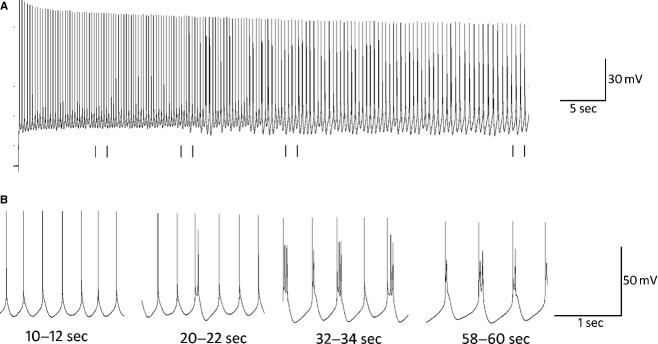
(A) Example trace recorded from a neuron in the presence of 10 μmol/L TCB‐2, 20 μmol/L DNQX, and 10 μmol/L bicuculline. This cell was held at −42 mV (threshold = −44 mV) for 60 sec by the steady injection of DC current. (B) Expanded sections of A from 10–12, 20–22, 32–34, and 58–60 sec are shown to demonstrate the evolution of rhythmic bursting throughout the trace. Tonic spiking is observed from 0 to 20 sec at which point single spikes are interspersed with doublets as the cell transitions to burst firing. From 30 sec to the end of the trace, the cell fires in a nearly‐exclusive bursting mode. Spike height and burst duration each decrease toward the end of the trace.

**Figure 2.**
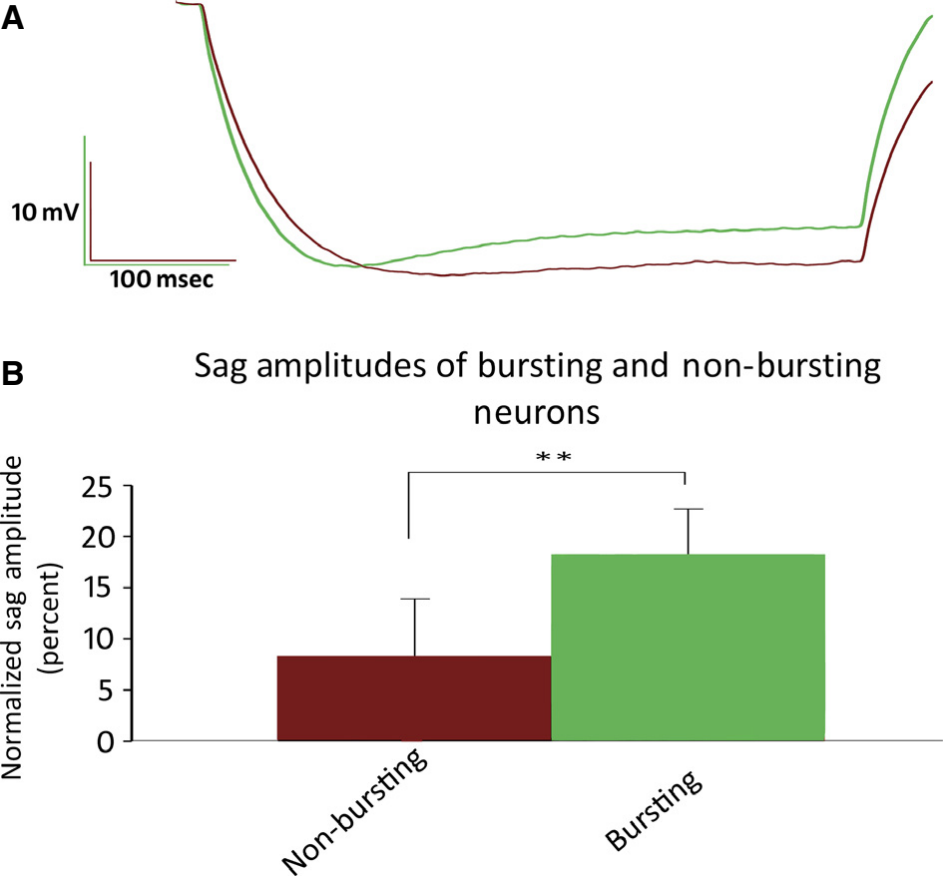
(A) Averaged traces recorded in response to a 450 msec 150 pA hyperpolarizing current injection. Traces from cells that fired ROB action potentials are depicted in green (*n* = 7), whereas cells that did not produce ROB are depicted in maroon (*n* = 7). (B) Normalized sag amplitudes of bursting and nonbursting cells. Sag amplitudes were calculated as the differences between the maximal hyperpolarization amplitudes and the amplitudes at 400 msec. Sag amplitudes are expressed as a percentage of the maximal hyperpolarization. Bursting neurons displayed significantly larger hyperpolarization‐mediated sags (18.2 ± 4.38%) than nonbursting neurons (8.30 ± 5.56%, *P* < 0.01 one‐tailed, unpaired *t*‐test).

To examine the effects of 5‐HT_2A_ activation on intrinsic properties of layer 5 pyramids, synaptic activity was blocked in a series of experiments with the AMPA antagonist DNQX (20 μmol/L) and the GABA_A_ antagonist bicuculline (10 μmol/L). In the presence of both DNQX and bicuculline, TCB‐2‐induced (10 μmol/L) recurrent oscillatory bursting (ROB) after 15–20 sec of tonic spiking evoked by steady state DC current injection (Fig. 1) in 7 of the 14 cells initially tested. On further examination we found that all cells in which ROB was induced exhibited a prominent depolarizing sag in response to a 450 msec 150 pA hyperpolarizing current (mean = 18.2 ± 4.4%; Fig. 2A), whereas cells that did not exhibit ROB produced significantly smaller voltage sags (mean = 8.3 ± 5.6%, *P* < 0.01 unpaired *t*‐test). To further characterize TCB‐2‐mediated bursting, all further experiments were conducted on neurons that displayed a minimal 12% depolarizing sag.

**Figure 3.**
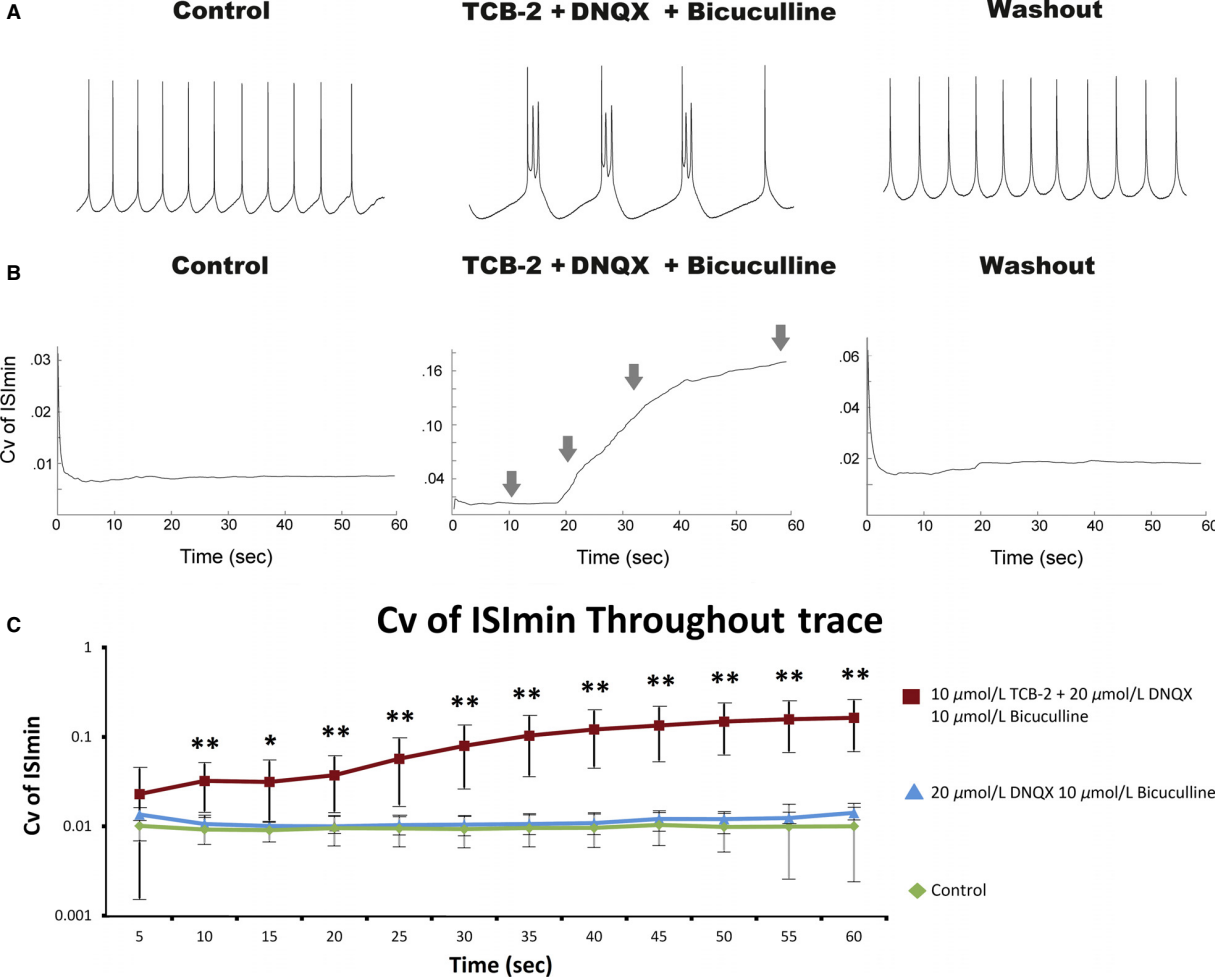
(A) Expanded sections (30–32 sec) from 60 sec traces recorded from the same cell depicted in Figure 1. All traces displayed were recorded from the same cell before, during application of, and after washout of 10 μmol/L TCB‐2, 20 μmol/L DNQX, and 10 μmol/L bicuculline. (B) Example curves of the coefficients of variation (σ/μ) of ISI_min_ calculated from a 6‐point moving average throughout 60 sec traces. Curves were generated from the same traces depicted in Figure 2A. Notice the difference in scale ranges between conditions (control 0–0.03, TCB‐2 + bicuculline and DNQX 0–0.18, washout 0–0.06). Arrows denote time of traces in Figure 1B. (C) Means of Cv curves sampled at 5 sec intervals (*n* = 7 cells). In the presence of TCB‐2, bicuculline, and DNQX, cells displayed significantly greater Cv of ISI_min_ than either control conditions or bicuculline and DNQX alone (paired *t*‐tests, *P < 0.05, **P < 0.01). Note that these data are plotted on a logarithmic scale. Error bars represent standard deviation.

It is interesting to note that ROB discharge was observed only by injecting current that brought the neurons to just suprathreshold membrane potentials; the mean current that elicited ROB was 116% of rheobase which elicited a stable spike rate of 3.8 ± 1.2 Hz. Increasing the amount of current injected resulted in higher frequency tonic spiking, but bursting was not observed at any time interval (data not shown as frequent, repeated applications of 60 sec suprathreshold currents compromised cell health and made the rigorous investigation of this phenomenon impossible). Under the conditions outlined above, bursts of action potentials were initiated at an interburst rate of 1.4 ± 0.3 Hz and displayed a mean intraburst firing rate of 26.6 ± 13.9 spikes/sec. This effect was reversible by washing out drugs with a 5–10 min rinse in aCSF (Fig. 3A).

### Variation of ISI_min_

Since ROB behavior evolved throughout time in these recordings, we needed a method to assess bursting over short time intervals throughout the trace. We determined that assessing bursting by calculating the Cv of ISI based on a 6‐point moving average was prone to unacceptable error, whereas calculating the Cv of ISI over intervals >6 points resulted in an unacceptably low sensitivity to burst detection. The alternate method we employed was to assess variation in the membrane potential between spikes. This method proved to be sufficiently sensitive to evaluate changes in firing mode with minimal error (Fig. 3B). A one‐way ANOVA (*F*_11,83_ = 5.32, *P* < 0.0001) with post hoc Tukey–Kramer tests indicated that the Cv of ISI_min_ was significantly greater at all times later than 40 sec than at 5 sec in the presence of TCB‐2 with DNQX and bicuculline. Neurons that were administered TCB‐2 during synaptic blockade displayed significantly higher Cv of ISI_min_ than control condition, or DNQX/bicuculline alone at all times later than 10 sec (Fig. 3C).

### Contributions of synaptic blockers and 5‐HT_2a_ receptor agonism

**Figure 4.**
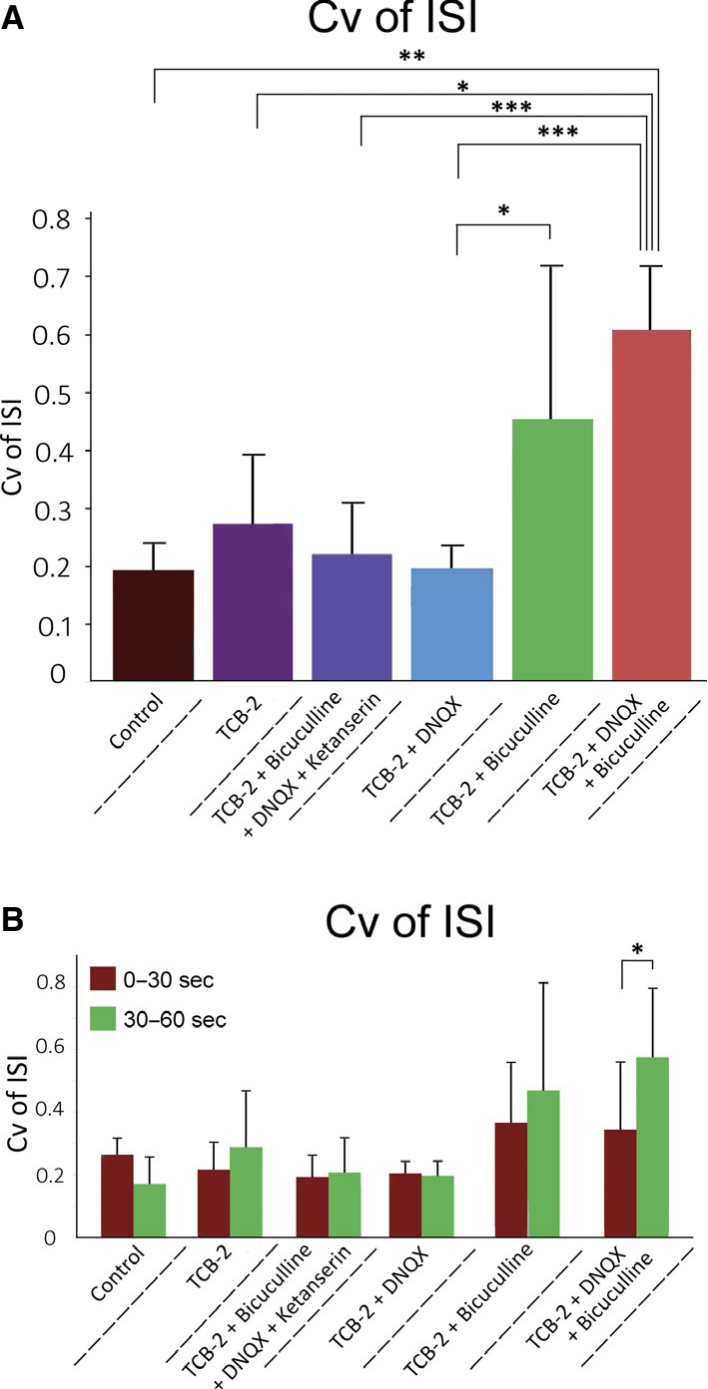
(A) Differences in Cv of ISI resulting from administration of various drug combinations. Cv was calculated from full 60 sec traces. Mean values and standard deviations of data represented in this figure are displayed in Table 1. One‐tailed, unpaired *t*‐tests were conducted between groups assuming unequal variance. Asterisks denote statistical significance: *P < 0.05, **P < 0.01, ***P < 0.001. (B) Cv of ISI calculated from same data depicted in A. For each treatment group, Cv is reported from 0 to 30 and 30 to 60 sec to demonstrate changes in Cv during recordings. The Cv in the first 30 sec (mean = 0.46 ± 0.21) recorded in cells perfused with TCB‐2, DNQX, and bicuculline was significantly lower than the Cv of the last 30 sec (mean = 0.69 ± 0.21, *P* = 0.036, paired *t*‐test).

**Figure 5.**
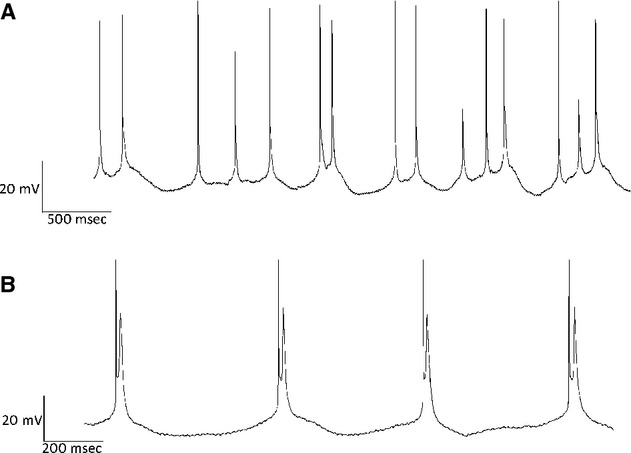
Examples of bursting observed in neurons that were treated with 10 μmol/L TCB‐2 and 10 μmol/L bicuculline. (A) Four second trace from neuron that exhibited arrhythmic bursting in response to a 60 sec current injection. Note that single spikes are interspersed with doublets and triplets. (B). A 2 sec trace depicting high‐frequency doublets. This behavior was seen in two different cells, intraburst frequency in both cases was >50 Hz.

**Figure 6.**
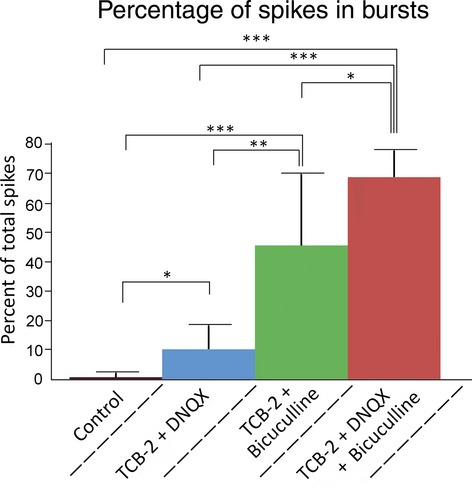
Percentage of spikes fired in bursts throughout 60 sec traces. Intraburst periods were calculated based on changes in ISI_min_ where burst initiations and terminations were classified as any periods where ISI_min_(*n* + 1) − ISI_min_(*n*) >2 mV and ISI_min_(*n* + 1) − ISI_min_(*n*) <−2 mV, respectively. Mean values for figures are: Control 0.7 ± 1.2, TCB‐2 + DNQX 10.2 ± 7.8, TCB‐2 + bicuculline 34.7 ± 33.5, and TCB‐2 + DNQX + bicuculline 68.7 ± 9.2. One‐tailed unpaired *t*‐tests were conducted between groups assuming unequal variance. Asterisks denote statistical significance: *P < 0.05, **P < 0.01, ***P < 0.001.

**Figure 7.**
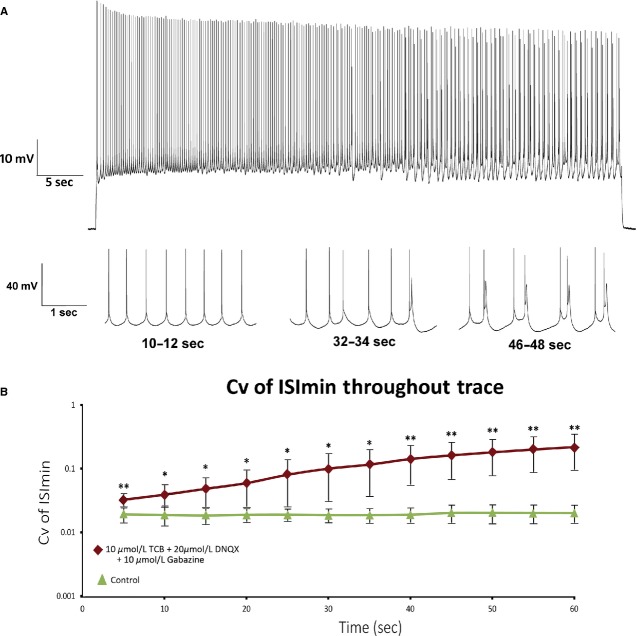
(A) Full 60‐sec trace recorded from neuron superfused with 10 μmol/L TCB‐2, 20 μmol/L DNQX, and 10 μmol/L gabazine with expanded sections from 10 to 12, 32 to 34, and 46 to 48 sec. ROB firing characteristics was very similar to that seen in cells treated with 10 μmol/L TCB‐2, 20 μmol/L DNQX, and 10 μmol/L bicuculline. (B) Cv of ISI_min_ curves generated from seven neurons were averaged at 5 sec intervals. In the presence of 10 μmol/L TCB‐2, 20 μmol/L DNQX, and 10 μmol/L gabazine, neurons exhibit significantly greater Cv of ISI_min_ than in a CSF at all times examined (*P < 0.05, **P < 0.01, paired *t*‐tests).

To determine the role of 5‐HT_2A_ receptor activation and the necessity of synaptic blockade for burst firing we calculated the Cv of ISI of neurons that were administered TCB‐2 alone, or TCB‐2 with either DNQX or bicuculline (Fig. 4, Table 1). Sixty second depolarizing current injections failed to elicit ROB discharge from neurons superfused with TCB‐2 alone or TCB‐2 with DNQX (this will be examined in more detail later). Of five neurons administered TCB‐2 with bicuculline, rhythmic bursting (high‐frequency doublets >100 Hz) was observed in one cell, arrhythmic bursting in two cells, and tonic firing in two cells (Fig. 5). We further examined the role of the 5‐HT_2A_ receptor in a subset of cells by the coapplication of 750 nmol/L of the 5‐HT_2A/2C_ antagonist ketanserin with DNQX, bicuculline, and TCB‐2. The depolarization observed after application of 10 μmol/L TCB‐2 was effectively blocked by the addition of 750 nmol/L ketanserin (control mean = −65.5 ± 3.0 mV, ketanserin with TCB‐2 and synaptic blockers mean = −63.5 ± 4.5 mV, *P* = 0.5, two‐tailed paired *t*‐test). Bursting behavior was not observed in neurons in the presence of ketanserin and Cv of ISI was not significantly different from controls (control mean = 0.203 ± 0.056, ketanserin with TCB‐2, DNQX, and bicuculline mean = 0.230 ± 0.090, *P* = 0.94, two‐tailed unpaired *t*‐test for unequal variance). While neurons administered TCB‐2 with bicuculline exhibited Cv of ISI that was not significantly different from neurons administered TCB‐2 with bicuculline and DNQX, the mean percentage of spikes that occurred within bursts throughout spike trains (Fig. 6) was significantly lower in the former group (mean = 45.5 ± 24.3% vs. mean = 68.8 ± 9.2%, *P* = 0.04 unpaired *t*‐test for unequal variance). To control for potentially confounding effects of bicuculline application (as bicuculline is known to antagonize several potassium channels), additional experiments were conducted in seven cells using the GABA_A_ antagonist gabazine. Similar to the results presented in Figure 3, 60 sec current injections in the presence of 10 μmol/L TCB‐2 with 20 μmol/L DNQX and 10 μmol/L gabazine consistently produced ROB (Fig. 7). There were no statistical differences between neurons superfused with TCB‐2, DNQX, and either bicuculline or gabazine by any of the metrics used in this study (intraburst spike rate = 23.8 ± 10.5 spikes/sec, *P* = 0.42; Cv of ISI = 0.62 ± 0.28, *P* = 0.65; percentage of spikes in bursts = 66 ± 12%, *P* = 0.67; all two‐tailed unpaired *t*‐tests for unequal variance).

**Table 1.** Summary of TCB‐2 induced alterations to Cv of ISI.

Drug treatment	*n*	Cv of ISI					
		Mean (whole trace)	SD	Mean (0–30 sec)	SD	Mean (30–60 sec)	SD
Control	7	0.203	0.056	0.208	0.064	0.165	0.066
TCB‐2	5	0.282	0.132	0.216	0.081	0.288	0.174
TCB‐2 + bicuculline + DNQX + ketanserin	5	0.230	0.090	0.193	0.064	0.206	0.106
TCB‐2 + DNQX	4	0.206	0.356	0.202	0.032	0.105	0.040
TCB‐2 + bicuculline	5	0.463	0.263	0.367	0.187	0.470	0.338
TCB‐2 + DNQX + bicuculline	7	0.617	0.207	0.461	0.212	0.686	0.214
TCB‐2 + DNQX + gabazine	7	0.622	0.28	0.401	0.246	0.772	0.390

Whole trace (60 sec), 0–30 sec, and 30–60 sec Cv of ISI for all drug combinations used in this study. Statistical comparisons between groups are depicted in fig. 4. The drug concentrations listed in this table are as follows: TCB‐2 10 μmol/L, bicuculline 10 μmol/L, DNQX 20 μmol/L, ketanserin 750 nmol/L, and gabazine 10 μmol/L.

### Stabilizing effect of inhibitory postsynaptic potentials

**Figure 8.**
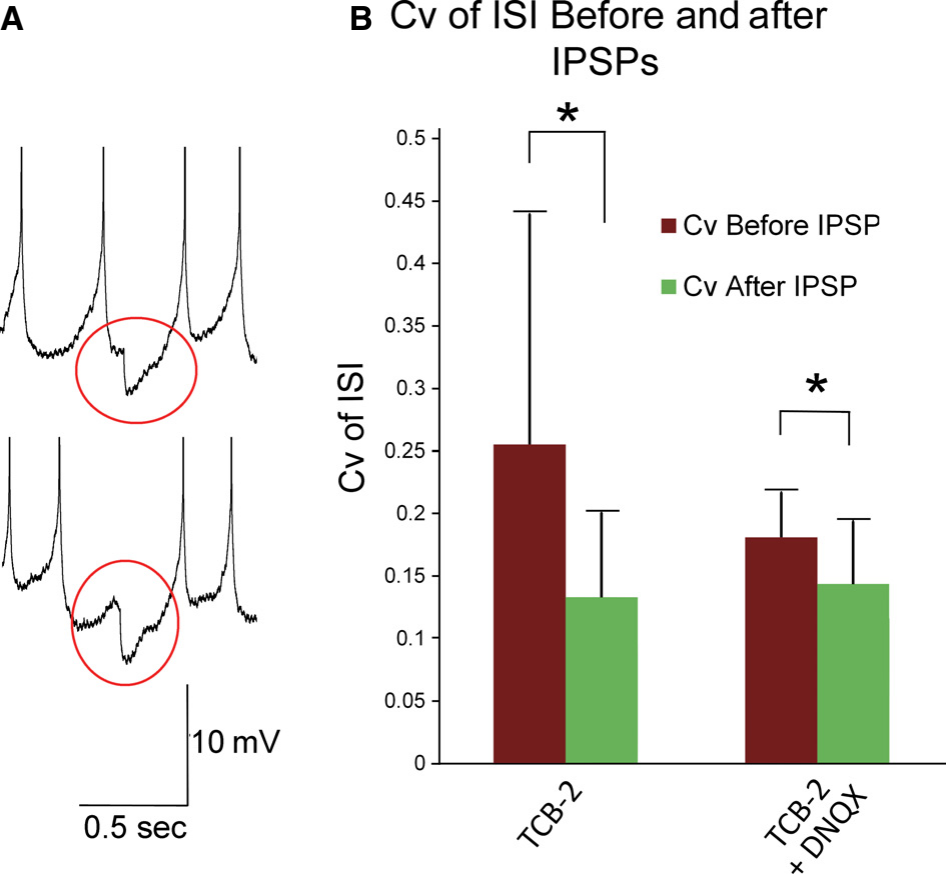
(A) Example traces to demonstrate visually identified IPSPs during a 60 sec induced spike train. Gray circles are used to highlight visually identified IPSPs. Spikes were truncated at −20 mV. (B) Cv of ISI was calculated based on the 4 ISIs before and after visually identified IPSPs. Twelve IPSPs from three different traces recorded from neurons in the presence of 10 μmol/L TCB‐2 and 14 IPSPs from three different traces recorded in 10 μmol/L TCB‐2 with 20 μmol/L DNQX were used. In 10 μmol/L TCB, the mean Cv before IPSPs was 0.26 ± 0.18 and the mean Cv after IPSPs was 0.14 ± 0.07, *P* = 0.036. In 10 μmol/L TCB‐2 with 20 μmol/L DNQX, the mean Cv before IPSPs was 0.18 ± 0.04, after IPSPs the mean Cv was 0.15 ± 0.05, *P* = 0.046. Paired *t*‐tests were used to examine both groups.

As previously mentioned, application of TCB‐2 alone or TCB‐2 with DNQX produced no substantial bursting and no significant changes to Cv of ISI (see Fig. 4). Closer examination of the data revealed large inhibitory postsynaptic potentials (IPSPs) with amplitudes as large as −10 mV in traces recorded in the absence of bicuculline. While recording data in real time, we found that in neurons held at suprathreshold potentials in the presence of either TCB‐2 or TCB‐2 with DNQX, ISI_min_ potentials would occasionally fluctuate as though the cell was entering burst firing mode, but large IPSPs between spikes appeared to restabilize the ISI_min_. To examine the possibility that IPSPs bias a neuron toward tonic spiking, we examined the Cv of ISI in the four interspike intervals prior to, and four intervals following visually identified IPSPs of at least −3 mV amplitude (e.g., see Fig. 8A). Fourteen IPSPs total were selected from three different traces recorded during application of TCB‐2 and DNQX, and 12 IPSPs were selected from three traces recorded during application of TCB‐2. In both cases the Cv of ISI was significantly lower in the four points following IPSPs than in the four points preceding them (Fig. 8B).

## Discussion

In summary, this study presents evidence that 5‐HT_2A_ receptor activation via the selective agonist TCB‐2 elicits recurrent oscillatory bursting in layer 5 pyramidal neurons of the medial prefrontal cortex, in the absence of excitatory and inhibitory synaptic transmission. Normalized I_h_ sag amplitudes were significantly correlated with ROB in neurons; bursting was only observed in cells that displayed 12% sag or greater. The substitution of bicuculline with the GABA_A_ antagonist gabazine did not affect burst profiles in seven cells tested which leads us to conclude that the ROB we observed was not caused by the actions of bicuculline on potassium ion channels. Burst discharge is suppressed by inhibitory synaptic activity, and only occurs after a 15–20 sec period of regular spiking. This effect was reversed by washout of TCB‐2 and prevented by application of the 5‐HT_2A/2C_ receptor antagonist ketanserin. Although the data presented here do not allow us to definitively conclude that 5‐HT_2A_ receptor activation causes ROB per se, we can confidently conclude that 5‐HT_2A_ receptor activation biases type I neurons toward firing in burst mode.

### 5‐HT_2A_ receptor mediated bursting

The 5‐HT_2A_ agonist DOI has previously been shown to increase the percentage of bursting in spike trains recorded in vivo in pyramidal neurons of the medial prefrontal cortex (Celada et al. 2008). Serotonin‐induced bursting has also been observed in vitro in pyramidal neurons of the electrosensory lateral line lobe of the weakly electric fish *A. leptorhynchus* (Deemyad et al. 2011). To our knowledge, this study presents the first evidence that activation of the 5‐HT_2A_ receptor elicits ROB discharge in pyramidal neurons of mammals in vitro. One possible reason that this phenomenon has not been previously observed is the time scale of the experiments we conducted. We held neurons at suprathreshold potentials for 60 sec, which is long enough for the inactivation of D‐type potassium channels (for review see Baranauskas 2007). Inhibition of the D‐current has been shown to promote bursting in pyramidal neurons in the CA1 region of the hippocampus of rats (Metz et al. 2007). Further research will be needed to determine the possible role that the D‐type K^+^ current plays in suppressing TCB‐2‐induced burst firing in the mPFC.

Results of our initial experiments in which bursting was only observed in 7 of the 14 neurons led to further analysis which revealed that recurrent bursting was predicted by the presence of a hyperpolarization‐activated voltage sag. This is particularly interesting as multiple studies which have examined differences between subpopulations of layer 5 pyramidal neurons have consistently found that subcortically projecting neurons (referred to as type I [Christophe et al. 2005], type A [Gee et al. 2012], or intrinsically bursting [Yang et al. 1996]) exhibit a pronounced I_h_‐mediated voltage sag, whereas callosally projecting neurons did not. Using retrograde tracing in conjunction with focal application of 5‐HT during electrophysiological recordings, Avesar and Gulledge (2012) found that differences in serotonergic excitability of layer 5 pyramidal neurons were correlated with differences in neuronal projection patterns as well as I_h_ mediated voltage sags. They found that neurons which exhibited either excitatory or biphasic responses projected callosally and did not display a sag in response to a hyperpolarizing current injection, whereas pontine‐projecting, I_h_‐displaying neurons were universally inhibited by 5‐HT application. This may seem at odds with our finding that application of TCB‐2 has an overall excitatory effect on these I_h_‐expressing pyramidal neurons (average 4.3 mV depolarization), but it is important to note that our study was focused on the actions of the 5‐HT_2A_ receptor rather than the overall actions of 5‐HT. The fact that we did not observe neuronal inhibition likely stems from the fact that we did not activate 5‐HT_1A_ receptors which are expressed on the axon initial segments of these neurons (see Puig and Gulledge 2011).

### Bursting is inhibited by synaptic activity

On the basis of the facts that bursting was only observed in the presence of bicuculline and that ISI variance was significantly reduced following visually identified IPSPs, we conclude that GABAergic neurotransmission inhibits bursting in single cells. Large‐amplitude IPSPs (>3 mV) that we included in analyses were only observed in the presence of TCB‐2. Even relatively small (1–2 mV) IPSPs were rarely observed in traces recorded in control aCSF. Application of the 5‐HT_2A_ agonist DOI has been shown to increase concentrations of GABA in the extracellular space of rat prefrontal cortices by the excitation of GABAergic interneurons (Abi‐Saab et al. 1999). A study by Puig et al. (2010) found that 5‐HT_2A_ receptors are expressed by approximately 30% of GABAergic parvalbumin‐positive fast spiking interneurons in layer 5 of the prefrontal cortex. These interneurons (axoaxonic or chandelier cells) synapse on the axon initial segments of layer 5 pyramidal neurons and thereby exert potent control over spiking behavior. As such, it is not surprising that Weber and Andrade (2010) found that application of the 5‐HT_2A_ agonist α‐methyl‐5‐HT caused a net depolarization of fast spiking interneurons as well as an increase in the frequency of IPSPs recorded in layer 5 pyramidal neurons.

Inhibitory postsynaptic potentials inhibition of burst activity may also reflect a more specific mechanism of burst regulation. IPSPs have been shown to regulate spiking behavior in single neurons (Williams and Stuart 2003) and are thought to play a prominent role in synchronizing networks of pyramidal neurons (for review see McCormick and Contreras 2001). It has also been hypothesized that low‐threshold‐spiking interneurons in layer 5 that synapse on the apical dendrites of layer 5 pyramidal neurons are capable of both detecting and regulating burst discharge in these neurons (Goldberg et al. 2004). By simulating IPSPs via current injection at different dendritic sites, Williams and Stuart (2003) found that the amplitude of IPSPs increased with increasing distance from the somas of layer 5 pyramidal neurons. Based on this observation, it is possible that low‐threshold‐spiking interneurons generate IPSPs in the distal apical dendrites of layer 5 pyramidal neurons, and that these IPSPs are amplified by active dendritic properties to produce large IPSPs similar to those we observed. More research is needed to determine both the source of these large IPSPs, and the mechanisms through which they inhibit bursting behavior.

It is also interesting that TCB‐2 with bicuculline did not consistently produce the same ROB seen with TCB‐2, bicuculline, and DNQX. Burst‐firing was seen in three of five cells that were administered TCB‐2 with bicuculline. This bursting was qualitatively different from that seen in the presence of DNQX, however. The bursting elicited by TCB‐2 and bicuculline generally consisted of arrhythmic bursts interspersed with single spikes. In one cell that exhibited rhythmic bursting, high‐frequency doublets (>100 spikes/sec) were initiated at 3 Hz which is also qualitatively different from the TCB‐2‐induced bursting recorded in the presence of DNQX (mean 26.6 spikes/sec that recurred at 1.4 Hz). High‐frequency doublets were also observed in cells that were administered TCB‐2 with DNQX and bicuculline, but these doublets generally occurred at the end of larger bursts similarly to those reported by Doiron et al. (2003). While we did not assess the effects of excitatory postsynaptic potentials explicitly, we suspect that glutamatergic activity destabilized the rhythmicity of bursts by introducing fluctuations in the membrane potentials of the recorded cells.

### Bursting elicited by dendritic stimulation

While the precise mechanisms that underlie TCB‐2‐induced bursting remain to be determined, it seems relevant to note the dense localization of 5‐HT_2A_ receptors throughout the somatodendritic compartments (including the apical dendrites) of layer 5 pyramidal neurons (Willins et al. 1997; Magalhaes et al. 2010; Weber and Andrade 2010). ROB discharge with similar profiles to what we report here has previously been reported following the focal application of glutamate to the apical dendrites of layer 5 pyramidal neurons of the rat sensorimotor cortex (Schwindt and Crill 1999). In their study, Schwindt and Crill found that stimulation of the apical dendrites alone could elicit bursting and that subthreshold current injection into the somas of cells increased the likelihood of burst discharge. Similarly Williams and Stuart (1999) and Larkum et al. (2004) each reported that burst discharge could be elicited by concurrent stimulation of the apical dendrites and somas of layer 5 pyramidal neurons. This mode of burst firing has been attributed to “back‐propagation‐activated Ca^2+^ spike firing” (BAC firing) (for review see Larkum 2013). It is conceivable that TCB‐2 may promote ROB by enhancing BAC firing.

This proposed mechanism underlying 5‐HT_2A_ receptor‐induced bursting remains debatable though, as Carr et al. (2002) found that activation of 5‐HT_2A_ receptors decreased the amplitude of backpropagating action potentials in rat layer 5 pyramidal cells of the medial prefrontal cortex, due to inhibition of the persistent sodium current (INaP). Their study found that the reduction in INaP was PKC dependent. These findings contrast with studies which have reported that PKC activation increases neuronal excitability by shifting the activation threshold of INaP to more hyperpolarized potentials in layer 5 pyramidal neurons of the somatosensory cortex (Astman et al. 1998) and dissociated pyramidal neurons of the sensorimotor cortex (Franceschetti et al. 2000).

Whether TCB‐2 induces bursting by increasing excitability of the apical dendrite or TCB‐2‐induced bursting relies on a novel mechanism remains to be determined and further work is needed to discover the mechanisms that underlie 5‐HT_2A_ receptor‐induced bursting. Irrespective of the specific mechanisms through which 5‐HT_2A_ receptor activation affects neuronal firing mode, if bursts of action potentials do in fact signal the convergence of information streams to downstream targets, then it is reasonable to suggest that abnormal stimulation of 5‐HT_2A_ receptors (either pharmacologically by psychotropic drugs or by 5‐HT_2A_ receptor dysregulation) could contribute to abnormalities in global information processing and thereby contribute to executive and perceptual functional abnormalities.

## Conflict of Interest

None declared.
